# Role of Glutamate Excitotoxicity in Glioblastoma Growth and Its Implications in Treatment

**DOI:** 10.1002/cbin.70005

**Published:** 2025-02-27

**Authors:** Colin Moriarty, Natasha Gupta, Debanjan Bhattacharya

**Affiliations:** ^1^ Department of Neurology and Rehabilitation Medicine University of Cincinnati College of Medicine Cincinnati Ohio USA

**Keywords:** cystine‐glutamate antiporter, GLAST, glioblastoma, glutamate, mGlu3R, temozolomide

## Abstract

Glioblastoma is a highly malignant and invasive type of primary brain tumor that originates from astrocytes. Glutamate, a neurotransmitter in the brain plays a crucial role in excitotoxic cell death. Excessive glutamate triggers a pathological process known as glutamate excitotoxicity, leading to neuronal damage. This excitotoxicity contributes to neuronal death and tumor necrosis in glioblastoma, resulting in seizures and symptoms such as difficulty in concentrating, low energy, depression, and insomnia. Glioblastoma cells, derived from astrocytes, fail to maintain glutamate‐glutamine homeostasis, releasing excess glutamate into the extracellular space. This glutamate activates ionotropic N‐methyl‐d‐aspartate (NMDA) receptors and α‐amino‐3‐hydroxy‐5‐methyl‐4‐isoxazolepropionic acid (AMPA) receptors on nearby neurons, causing hyperexcitability and triggering apoptosis through caspase activation. Additionally, glioblastoma cells possess calcium‐permeable AMPA receptors, which are activated by glutamate in an autocrine manner. This activation increases intracellular calcium levels, triggering various signaling pathways. Alkylating agent temozolomide has been used to counteract glutamate excitotoxicity, but its efficacy in directly combating excitotoxicity is limited due to the development of resistance in glioblastoma cells. There is an unmet need for alternative biochemical agents that can have the greatest impact on reducing glutamate excitotoxicity in glioblastoma. In this review, we discuss the mechanism and various signaling pathways involved in glutamate excitotoxicity in glioblastoma cells. We also examine the roles of various receptor and transporter proteins, in glutamate excitotoxicity and highlight biochemical agents that can mitigate glutamate excitotoxicity in glioblastoma and serve as potential therapeutic agents.

**Abbreviations**
ALT: alanine aminotransferase
AMPAR: α‐amino‐3‐hydroxy‐5‐methyl‐4‐isoxazolepropionic acid receptor
AST: astaxanthin
BCAAT: branched‐chain amino acid transaminases
BDNF: brain‐derived neurotrophic factor
COX2: cyclooxygenase‐2
CREB: cAMP response element binding protein
DMFO: difluoromethylornithine
EAAT: excitatory amino acid transporter
FADD: fas‐associated death domain
GBM: glioblastoma multiforme
GDH: glutamate dehydrogenase
GLAST: glutamate‐aspartate transporter
GLS: glutaminase
GPx: glutathione peroxidase
GS: glutamine synthetase
GLT‐1: glutamate transporter 1
GSC: gliblastoma stem cell
HIRMAb: human insulin receptor monoclonial antibody
IDH1: isocitrate dehydrogenase 1
IDH2: isocitrate dehydrogenase 2
KD: ketogenic diet
MAPK: mitogen‐activated protein kinase
MCP‐1: monocyte chemoattractant protein‐1
MGMT: methylguanine methyltransferase
MSO: methionine sulfoximine
NAD: nicotinamide adenine dinucleotide
NMDA receptors: N‐methyl‐d‐aspartate receptors
ROS: reactive oxygen species
SAT1: system A Transporter 1
SIRT: sirtuin
SN1 receptor: system N1 receptor or System N glutamine transporter
SNAT1: sodium‐coupled neutral amino acid transporter 1
SNAT2: sodium‐coupled neutral amino acid transporter 2
TMZ: temozolomide
TNF: tumor necrosis factor
TNF‐α: tumor necrosis factor alpha
TrkB: tropomyosin receptor kinase B

## 1 Introduction

Glioblastoma (also called GBM) is a malignant grade IV astrocytoma, which develops in the cerebral cortex of the brain. It is one of the most common yet highly invasive brain tumors accounting for more than 60% of all gliomas within the brain (Hanif et al. 2017). GBM patients have an overall 5‐year survival of 6.8%, making it a tumor with one of the worst prognoses in the entire spectrum of oncology. Glioblastoma tumors can disrupt neural signaling through several different mechanisms, most notably through glutamate excitotoxicity. Glutamate excitotoxicity is a process of neuronal cells death due to excessive activation of glutamate receptors. The primary mechanism by which glioblastoma cells cause glutamate excitotoxicity is through the excessive activity of the cysteine‐glutamate antiporter system Xc protein (SXc) complex located on their cell membranes. This antiporter causes an influx of extracellular cystine (a form of cysteine) into the GBM cell while simultaneously exporting glutamate into the extracellular environment in a 1:1 exchange ratio (Robert and Sontheimer 2014). With the use of this transporter, GBM cells increase extracellular glutamate concentrations which, in turn, activate N‐methyl‐d‐aspartate (NMDA) receptors on proximal neurons leading to an increase in calcium concentrations and the formation of reactive oxygen species (ROS), eventually leading to cell death (Hanif et al. 2017). In the glioma‐surrounding tissue, extracellular glutamate levels are elevated up to 100 times higher than in unaffected brains (Marcus et al. 2010; Roslin et al. 2003). The intracellular cysteine is then utilized to synthesize glutathione (GSH), an antioxidant that promotes GBM cell survival (Hanif et al. 2017).

High levels of glutamate add to the GBM pathology by stimulating the proliferation and invasion of glioma cells. Additionally, in surrounding neurons, glutamate‐driven hyperexcitation contributes to tumor growth by inducing excitotoxicity. This process leads to apoptosis in the affected neuronal cells creating room for the tumor to grow (Savaskan et al. 2015).

Alternatively, GBM cells can also cause glutamate excitotoxicity through the glutamine‐glutamate cycle (Natarajan and Venneti 2019). In this cycle, GBM cells uptake glutamate to produce glutamine, which is then transported to neurons to resynthesize glutamate.

Glutamate excitotoxicity is enhanced when there is an increase in the intracellular Ca^2+^ level. Specifically, there is a positive feedback mechanism that increases calcium intracellularly while increasing glutamate extracellularly through excitotoxicity (Pei et al. 2020). Therefore, inhibiting the calcium influx in GBM cells can be a promising therapeutic option. Current therapeutic options to prevent glutamate excitotoxicity include alkylating agents that target the gene that produces the human SX_c_ antiporter system, most notably temozolomide (TMZ) and sulfasalazine. However, GBM cells have developed resistance to many of these therapies by activating various pathways and receptors, including mechanisms of DNA repair. Therefore, it is important to identify other sources causing excitotoxicity and to find ways to inhibit these pathways.

### 1.1 GBM Receptor and Transporter Proteins

GBM cells possess many different types of receptors which work toward their survival and growth, including the following: ionotropic α‐amino‐3‐hydroxy‐5‐methyl‐4‐isoxazolepropionic acid (AMPA), NMDA, and kainate glutamate receptors, group I‐III metabotropic receptors; excitatory amino acid transporters (EAATs), the cysteine‐glutamate antiporter (SXc) system, GABAA receptor, acid‐sensing ion channels (ASICs), Inward rectifying potassium channels, glutamate‐aspartate transporters (GLAST), and sodium‐coupled neutral amino acid transporters (SNAT) receptors (Natarajan and Venneti 2019). These receptors all work together in GBM cells to maintain a consistent resting membrane potential which aids in cell survival and functioning. In addition, GBM cells express the ClC‐3 channel which aids in regulating the intracellular calcium concentrations (Molenaar 2011). The AMPA, NMDA, and kainate receptors play a role in GBM progression by increasing the amount of intracellular calcium when activated (Natarajan and Venneti 2019). The type I‐III metabotropic receptors activate signaling pathways, such as the MEK and PI3K pathways, which promote GBM proliferation. The receptors that have a direct effect on glutamate excitotoxicity include: the SXc receptor, which has the most significant impact by releasing glutamate to the extracellular environment, and the GLAST receptor, which plays a key role in the glutamate‐glutamine cycle. A notable feature of GBM cells is the absence of the glutamate transporter GLT‐1, which prevents the uptake of glutamate into the cell and allows it to accumulate in the extracellular space (Brown et al. 2022). This excess glutamate can contribute to excitotoxicity, which leads to damage of surrounding healthy brain cells and exacerbates tumor progression. The lack of this transporter aids GBM cells in resisting radiation therapy and chemotherapy, promoting cell survival (Martins et al. 2020). As per prior studies, GLAST receptor is expressed in human glioma specimens in moderate to high levels, especially in high‐grade glioma samples and negative to low levels in low‐grade glioma samples in more than 50% of GBM cases (Corbetta et al. 2019).

The invasiveness of glioblastoma is linked to the glutamate release and expression of GLAST Magnetic spectroscopy has demonstrated that glutamate levels are increased in GLAST‐expressing gliomas compared to GLAST‐depleted gliomas. Furthermore, GBM stem‐like cells (GSCs) express GLAST receptors. However, despite expressing higher levels of GLAST glioblastoma stem cells (GSCs) release glutamate rather than taking it up and this significantly enhances their invasiveness (Corbetta et al. 2019).

Human GBM cells lack the expression of excitatory amino acid transporters‐2 (EAAT2), the human homolog of glutamate transporter 1 (GLT1). Additionally some GBM cells also lack the expression of EAAT1, the human equivalent of GLAST. These excitatory amino acid transporters (EAAT) are typically involved in glutamate clearance and the lack of expression of these receptors in GBM cells enhances the concentration of glutamate in the surrounding extracellular environment. GBM cells release glutamate through a cysteine‐glutamate exchanger (system xc^–^) protein which is upregulated in GBM cells. Enhanced activity of this exchanger system actively releases glutamate into the extracellular environment in exchange of uptake of cystine into the GBM cells contributing to glutamate excitotoxicity (Biegański and Szeliga 2024). This process is further exacerbated by the downregulated expression of glutamate transporters like GLT‐1, in GBM cells which limits the re‐uptake of extracelluar glutamate into the GBM cells.

### 1.2 The Glutamine‐Glutamate Cycle and Glutamate Excitotoxicity

**Figure 1:**
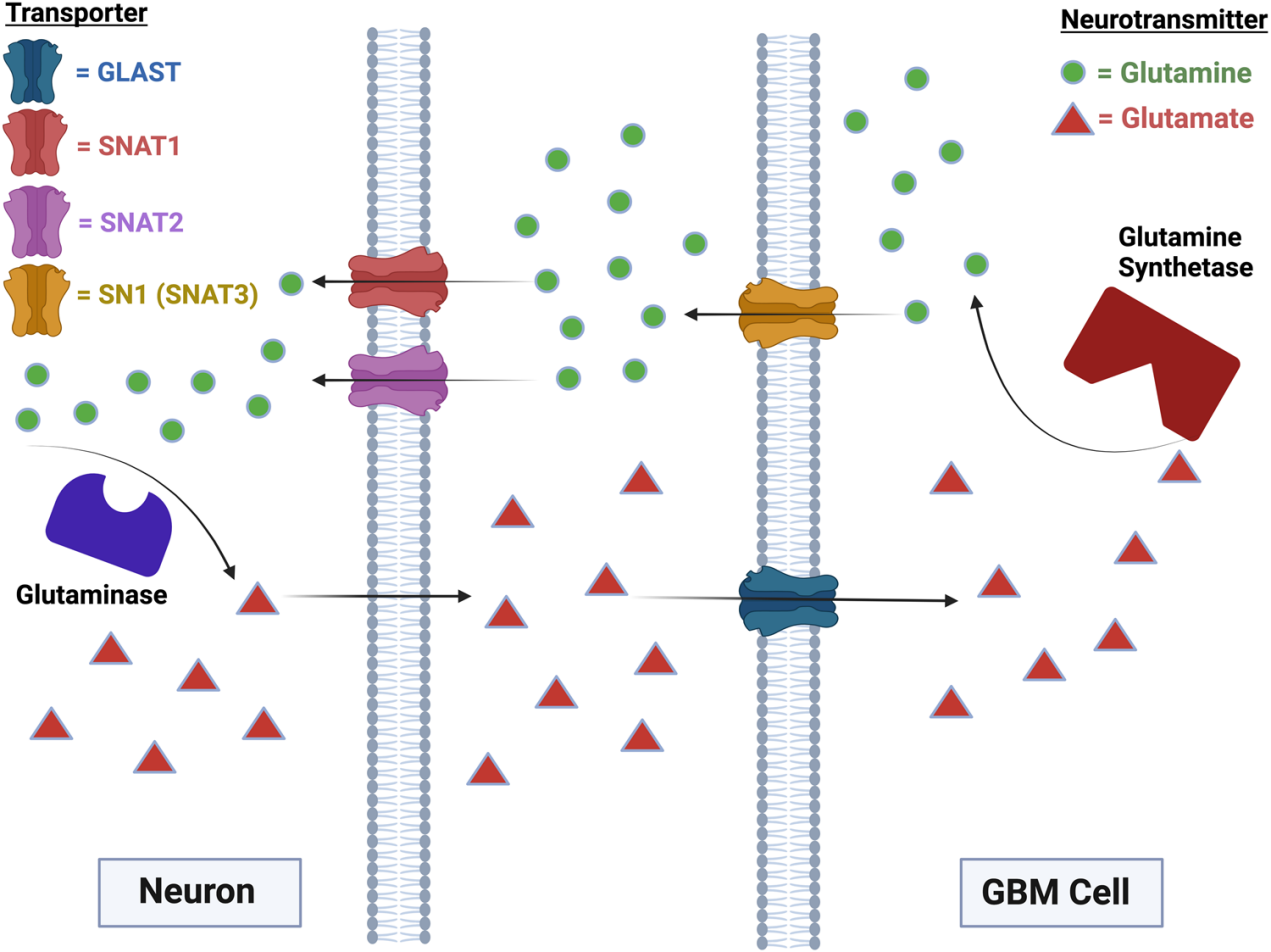
Glutamine‐Glutamate cycle between a neuron and a glioblastoma cell, facilitates excessive glutamate accumulation within the extracellular environment. This cycle manipulates neurons by inducing glutamate production by glutaminase, which is then fed to other proximal neurons. GBM cells take up glutamate via GLAST transporter. Inside GBM cells glutamate is converted into glutamine by glutamine synthetase. GBM cells release glutamine into the extracellular space which neurons take up via SNAT1 and SNAT2 and convert back to glutamate. GLAST, glutamate‐aspartate transporter; SNAT1, sodium coupled neutral amino acid transporter 1; SNAT2, sodium coupled neutral amino acid transporter 2; SN1 Receptor, System N glutamine transporter or System N1. Created with BioRender.com

The glutamine‐glutamate cycle between neurons and astrocytes is a way to maintain equilibrium between concentrations of glutamine and glutamate so that glutamine can be utilized properly for the synthesis of amino acids, nitrogenous bases, and NAD, as well as for the maintenance of cognitive functioning. This cycle works when neurons take up glutamine through an SNAT1 transporter and convert it to glutamate using glutaminase, producing ammonia as a by‐product (Figure 1). The converted glutamate is then transported to an astrocyte (or GBM cells) through a Na^+^/K^+^‐ATPase‐dependent ‐transporter protein, GLAST expressed on the membrane of GBM cells and converted to glutamine again by glutamine synthetase (Figure 1). This glutamine is then transported through sodium‐coupled neutral amino acid transporters (SNAT1) on astrocytes and gets released once again to neurons to continue the cycle (Martins et al. 2020; Obara‐Michlewska and Szeliga 2020). GBM cells are aggressive types of astrocytoma, and possess the ability to engage in this glutamine‐glutamate cycle and promote neuronal synthesis of glutamate, thereby exposing other surrounding neurons to high glutamate concentrations (Figure 1). Previous research has shown that depriving cancer cells of glutamine increases the expression of tumor suppressor protein p53 (Thomas et al. 2013). This, in turn, upregulates the synthesis of other arginine transporters, such as SLC1A3 and SLC7A2, which compensate for glutamine deficiency and promote GBM cell survival by activating the mTOR pathway (Xu et al. 2023).

Therefore, a potential therapeutic strategy could involve blocking or downregulating glutamine transport to prevent glioblastoma cells from compensating for glutamine deficiencies. This could further inhibit their ability to activate survival pathways.

The Glutamate‐aspartate transporter is highly expressed on the GBM cell plasma membranes. However, previous studies have shown that despite having this expression, glutamate uptake by GBM cells remained low due to their lack of expression of functional Na^+^/K^+^‐ATPase. Therefore, studies have attempted to overexpress Na^+^ /K^+^‐ATPase on GBM cells. The results showed increased glutamate uptake by the GBM cells and induced GBM cell apoptosis (Corbetta et al. 2019). Furthermore, other studies have also attempted to target GLAST expression in GBMs using UCPH‐101, a GLAST inhibitor. Results from the study showed that injecting UCPH‐101 in mice bearing GBM tumors significantly increased their survival. This intervention decreased GLAST expression and induced GBM cell apoptosis (Corbetta et al. 2019). Therefore, targeting glutamine transport by inhibiting GLAST receptors in GBMs might be a promising therapeutic intervention.

In addition, previous studies have shown that gliomas have a 3–5 times stronger expression of SNAT receptors in GBM tissue when compared to control brain tissue (Sidoryk et al. 2004). Studies have shown that silencing SNAT receptors in glioblastomas to reduce glutamine uptake by proximal neurons has not been effective as a standalone therapy. Glutamine transport and GBM proliferation remained unchanged (Sidoryk et al. 2006). Current literature investigating therapies that involve a combination of SNAT transporter silencing along with the silencing of other receptors involved in glutamine transport remains limited. Therefore, it is possible that silencing SNAT receptors in conjunction with other glutamine receptors may lead to an enhanced synergistic effect, which should be further explored. High expression of SLC transporters in GBM has been strongly correlated with poorer prognosis, making them potential targets for glioblastoma treatment (Anagnostakis et al. 2023). Previous studies on glioma treatment have attempted to target receptors in the SLC transporter family using agents such as difluoromethylornithine (DMFO), an inhibitor of ornithine decarboxylase 1, and AMXT 1501, a polyamine transporter inhibitor (Khan et al. 2021). Such therapy was shown to reduce polyamine uptake by gliomas and to improve survival in animal models by inducing apoptosis (Khan et al. 2021). Therefore, targeting amino acid transport through the SLC transporter system could be a promising targeted therapy for treating GBM patients.

## 2 Excitatory Amino Acid Transporters (EAATs) and Cystine‐Glutamate Antiporter (SXc) Gene Regulation on Glutamate Excitotoxicity

Extracellular glutamate concentration is significantly increased when EAAT genes are downregulated and SXc antiporter genes are upregulated in GBM (Robert and Sontheimer 2014). This mechanism allows GBM cells to intake more cystine and release intracellular glutamate, limiting the reuptake through downregulated EAATs. This causes reduced chance of glutamate excitotoxicity within GBM cells and increased excitotoxicity and cell death of neurons within the environment. In addition, the cysteine is converted to glutathione which assists in GBM survival. Excitotoxicity is influenced by the expression of EAATs on surrounding neurons. Upregulation of these transporters increases glutamate uptake, which can lead to neuronal apoptosis. GBM cells aim to regulate both glutamate and glutathione levels by coordinating the activity of these transporters (Robert and Sontheimer 2014).

### 2.1 Signaling Pathway Responses to Glutamate Excitotoxicity in Neuronal and GBM Cells

When neurons are exposed to a high level of glutamate, they activate the MAPK (Mitogen‐Activated Protein Kinase)/ERK pathway which helps them survive against excitotoxicity. This signaling works when brain‐derived neurotrophic factor (BDNF) binds to tropomyosin receptor kinase B (TrkB) which then activates the MAPK pathway. Once activated MAPKs like ERK (Extracellular signal Regulated Kinase) can directly phosphorylate cAMP response element binding protein (CREB), at specific serine residues. Activated CREB binds to cAMP response elements (CRE) in the promoter regions of target genes and can downregulate pro‐apoptotic factors and upregulate transcription of anti‐apoptotic factors such as Bcl‐2 thereby promoting neuronal survival (Ortuño‐Sahagún et al. 2014). Therefore, agonists that aid in stimulating this MAPK/ERK pathway can improve neuronal survival against excitotoxicity. Previous studies have shown that imipramine, a tricyclic antidepressant, favors this pathway, leading to neuronal survival in rat hippocampus' when exposed to high concentrations of glutamate (Ortuño‐Sahagún et al. 2014).

Furthermore, GBM cells can respond to extracellular glutamate through an autocrine signaling mechanism involving AMPA receptors (AMPAR) (van Vuurden et al. 2009). These receptors which are Ca^2+^ permeable are activated when glutamate binds to them. This activation increases intracellular calcium concentrations, which in turn triggers the activation of the protein kinase B (Akt) pathway (So et al. 2021). This signaling could potentially be disrupted for treatment as GBM cells could be targeted for insertion with a viral vector containing genes for Ca^2+^ impermeable AMPAR to prevent the calcium signaling. This signaling is typically initiated by Ca^2+^ permeable AMPAR, which activates the Akt pathway. Such a treatment could potentially decrease glioblastoma growth and mitigate drug resistance. Since GBM cells would be directly targeted in this method, surrounding neurons would likely be unharmed.

Furthermore, when Akt is dephosphorylated by a tumor suppressor protein called Phosphatase and tensin homolog (PTEN) the Akt pathway becomes inactivated, reducing the production of anti‐apoptotic factors and promoting cell death (Noch and Khalili 2009). In fact, previous studies have shown that low PTEN expression resulted in increased GBM proliferation and invasion, resulting in a worse prognosis (Hashemi et al. 2023). Furthermore, GBM cells with PTEN deletion tend to have increased resistance to chemotherapy. Therefore, upregulating PTEN as a way of downregulating Akt can help trigger necrosis in GBM cells and increase tumor cells' response to therapy.

## 3 GBM Tumor Necrosis From Glutamate Excitotoxicity

Glutamate can build up in the extracellular space around GBM cells when the function of excitatory amino acid transporter 2 (EAAT2), primarily responsible for extracellular glutamate uptake in the brain cells, is disrupted (Noch and Khalili 2009). This accumulation of glutamate inhibits the SXc system resulting in decreased intracellular glutathione production and, in turn, decreased resistance of GBM cells. Following mitigated resistance of GBM cells, glutamate ionotropic receptors become activated leading to a massive influx of calcium, the formation of ROS, and the depletion of ATP. This depletion causes sodium and water influx and inhibits potassium efflux causing the GBM cell to swell and rupture (Noch and Khalili 2009). One important feature of necrotizing GBM cells is their expression of EAAT2 receptors. These receptors facilitate the uptake of extracellular glutamate into the cells taken into the cell where it becomes a part of the intracellular glutamate pool. The SXc antiporter relies on the glutamate supplied by the EAAT2 to exchange for extracellular cystine. This cystine is crucial for synthesis of intracellular glutathione, and antioxidant essential for cell survival. A possible therapeutic intervention to increase necrosis in GBM cells could utilize RNA splicing technology, such as CRISPR/Cas9, to target the EAAT2 gene in GBM cells. Such therapeutic gene editing has been utilized with other genes in GBM cells (Begagić et al. 2024). Furthermore, GBM cells also possess sodium‐glutamate symporters and blocking the binding of sodium could potentially raise the extracellular glutamate concentrations and prevent further synthesis of glutathione (GSH). This second approach may reduce the production of glutathione, which supports GBM cell survival. However, it may also expose nearby neurons to high extracellular glutamate concentrations, which can cause neuronal cell death and potentially lead to tumor expansion.

### 3.1 Alkylating Agents as Therapies Against GBM

The most common therapeutic approach is applying alkylating agents to these cells to cause double‐stranded DNA breaks and favor apoptosis. TMZ is the most commonly used alkylating agent that causes double‐stranded breaks and triggers apoptosis. TMZ exerts its antineoplastic activity mainly through the process of genome‐wide DNA methylation. TMZ breaks the DNA chains by introducing a methyl group to guanine at the oxygen‐6 (O6), nitrogen‐7 (N7), and adenine‐3 (N3) sites resulting in the cytotoxic DNA lesions O6‐methylguanine (O6‐MG), N7‐methylguanine (N7‐MG), and N3‐methyladenine (N3‐MA) (J. Zhang et al. 2012). O6‐methylguanine (O6‐MG) is the most toxic DNA modification triggering apoptosis in glioma cells. During DNA replication, these lesions cause base pair mismatches, leading to cell‐cycle arrest in the G2/M phase and subsequent apoptosis (Jiapaer et al. 2018). However, GBM cells can resist this chemotherapeutic effect and continue to accumulate within the brain as they possess mechanisms of DNA repair. The common enzyme responsible for resistance against these types of treatments is Methylguanine methyltransferase (MGMT), which removes the methylation from the DNA caused by TMZ (Almeida Lima et al. 2023). Cells with higher MGMT levels tend to display greater resistance to TMZ in comparison to cells with lower concentrations. Resistance to TMZ in GBM cells is also promoted when they signal for ferroptosis, a process initiated by transcription factor NRF2 through which lipid peroxidation occurs. Sensitivity to TMZ is favored when *NRF2* is downregulated because this factor promotes the synthesis of DNA repair enzymes such as MGMT (Almeida Lima et al. 2023). TMZ sensitivity can be potentiated when combined with a GSH synthase inhibitor as blocking the production of GSH promotes TMZ to cause apoptosis.

Table 1 summarizes some of the currently available pharmacological agents (including alkylating agents) and therapies for managing GBM, detailing their respective targets, mechanisms of action, effects, and limitations.

**Table 1:** Summary of drugs currently used in management of glioblastoma and their targets, associated mechanisms of action, effects and limitations.

Treatments and Chemical agents	Targets	Mechanism of action	Effects	Limitations	Reference
Temozolomide (TMZ)	DNA, specifically the O6 and N7 positions of guanine residues	Through genome wide DNA methylation as it adds methyl groups to guanine residues in DNA, leading to DNA damage that triggers apoptotic cell death.	Prolongs survival and delays tumor progression when administered in combination with radiation therapy.	Limited efficacy in MGMT unmethylated glioblastoma patients. Resistance develops with high expression of MGMT which repairs TMZ‐induced DNA damage. Toxicity including myelosuppression inducing lymphopenia.	(J. Zhang et al. (2012); Karachi et al. (2018))
Bevacizumab (Avastin)	Vascular endothelial growth factor (VEGF)	A monoclonal antibody that inhibits angiogenesis by targeting VEGF and reducing blood flow to the tumor.	Reduces tumor‐associated edema and improves the quality of life. Short‐term control of tumor progression in some patients.	Treatment resistance develops due to the activation of compensatory angiogenesis pathways. Do not significantly improve overall survival.	(Sharma et al. (2019); Chinot et al. (2014))
Corticosteroids, e.g. Dexamethasone	Tumor associated edema and inflammation	Reduces peritumoral inflammation and cerebral edema DEX ameliorated cerebral edema by reducing the permeability of blood‐brain barrier, through regulating the expression level of occludin, claudin, and vascular endothelial (VE)‐cadherin thereby improving symptoms.	Symptomatic relief from increased cerebral edema and inflammation.	Does not target tumor cells directly and may be linked to poorer outcomes. Long‐term use causes immunosuppression.	(Dubinski et al. (2018); Kostaras et al. (2014); Zhou et al. (2021))
Sulfasalazine	SXc antiporter system	Depletes glutathione (GSH) levels, promoting ferroptosis in glioblastoma cells.	Blocks growth of tumor. Sulfasalazine sensitizes gliomas to gamma knife radiosurgery.	Multiple side effects Limited access to the blood‐brain barrier. Does not show any added benefit when combined with temozolomide.	(Sehm et al. (2016))
Lomustine (N‐chloroethyl‐N‐cyclohexyl‐nitrosourea, CCNU)	DNA (alkylation of DNA strands)	An alkylating agent that adds reactive metabolite chloroethyl groups to the DNA crosslinking of DNA casing tumor cell death.	Sometimes used in combination with temozolomide as a second‐line treatment. Used in TMZ‐resistant recurrent glioblastoma. Lomustine is sometime combined with procarbazine and vincristine in the PCV regimen to treat recurrent glioblastoma.	High recurrence after treatment and activity is restricted to MGMT promoter‐methylated glioblastoma. Hematological toxicity notably thrombocytopenia and leukopenia.	(Weller and Le Rhun (2020); Yamamuro et al. (2021); Kaina (2023); Schmidt et al. (2006))
Procarbazine	DNA (alkylation of DNA strands)	An alkylating agent, that inhibits DNA, RNA, and protein synthesis. DNA damage by hydrogen peroxide, formed during auto‐oxidation of procarbazine. Inhibit transmethylation of the methyl groups of methionine into t‐RNA. Lack of functional t‐RNA inhibits t‐RNA could cause cessation of protein synthesis, and consequently DNA and RNA synthesis.	Procarbazine is combined with lomustine (CCNU) and vincristine in the PCV regimen to treat primary and recurrent glioblastoma.However, combining the PCV regimen with radiaton therapy can only slightly extend the median survival.	Causes myleosuppression (leucopenia, thrombocytes). and neurological issues like headache, dizziness, confusion. It cannot improve overall survival as a standalone treatment in the PCV regimen.	(Weller et al. (2021))
Tumor Treating Fields (TTF) therapy, Optune	Mitotic spindle apparatus and microtubules	Noninvasive treatment which delivers low‐intensity alternating electric fields that disrupt cell division and induce apoptosis in dividing tumor cells.	Extends progression‐free survival when used with temozolomide.	Do not change overall survival. Patient needs to wear this device on head continuously at least 18 h a day which can be uncomfortable. Low adherence for some patients as they do not use this continuously due to the discomfort and lifestyle constraints This reduces its therapeutic benefits.	(Burri et al. (2018))
Durvalumab (PD‐L1 antibody)	PD‐L1	Durvalumab is human IgG monoclonal antibody that binds to PD‐L1 and CD80, and allowing T cells to recognize and kills tumor cells.	When combined with radiation marginally increase the progression free survival.	Failed to improve overall outcome in recurrent and newly diagnosed glioblastoma patients.	(Nayak et al. (2022))
Conventional radiation therapy	Targets cellular DNA	Ionizing radiation using photons (X‐rays) introduce DNA damage and activate cell death pathways.	When conventional radiation therapy is combined with concomitant Temozlomide it improves both overall and progression‐free survival.	Development of radio‐resistance leading to recurrence. Causes radiation toxicity including neurocognitive complications.	(Stupp et al. (2005))
Proton beam therapy (PBT)	Targets cellular DNA	Charged proton ions kill tumor cells by inducing breaks in the DNA double strand in tumors cells. Protons offer significant dosimetric benefits.	It provides more precise and focused delivery of radiation dose to tumors. Proton beam therapy the offers longer median of progression‐free survival that conventional radiation therapy. Significantly improves overall survival than patients receiving conventional radiation therapy. Neurocognitive complications of brain irradiation can be alleviated by proton beam therapy.	Although less than conventional radiation therapy, proton bean therapy causes grade 1 or grade 2 toxicity. Radiation necrosis as a late radiation‐related toxicity in high‐dose proton beam therapy.	(Matsuda et al. (2023); Das et al. (2024); Aiyappa‐Maudsley et al. (2022))

Furthermore, it has been discovered that U87 IDH mutant GBM cells respond to TMZ by increasing glutamate production from glucose stores instead of glutamine through the upregulation of pyruvate dehydrogenase (PDH) while decreasing β‐oxidation of fatty acids (Pușcaș et al. 2024). U87 cell line also responds to TMZ by increasing the activity of alanine aminotransferase (ALT), aspartate transaminase (AST), and glutamate dehydrogenase (GDH), all of which favor the synthesis of glutamate from α‐ketoglutarate (Subramani et al. 2020). These findings indicate that TMZ causes a change in GBM metabolic pathways and glutamate can be used as a biomarker to assess the resistance of GBM cells to TMZ (Subramani et al. 2020). This is especially beneficial for GBM IDH mutant types because they lack the enzymes required for the TCA cycle to function.

### 3.2 Role of Glutamate Excitotoxicity in Radiation Sensitivity of GBM Cells

Ionizing radiation (IR), in the form of gamma rays or X‐rays, is commonly used in the treatment of GBM to induce DNA damage and increase intracellular ROS concentrations (Yang et al. 2021). Ionizing radiation affects glutamate excitotoxicity by preventing glucose uptake in GBM cells, forcing them to rely on glutamine metabolism so they can generate energy for continued survival (Yang et al. 2021). Specifically, it upregulates glutaminase and glutamate dehydrogenase, which results in the conversion of glutamate to α‐ketoglutarate, which is then fed into the TCA cycle for excessive ATP production. When high amounts of α‐ketoglutarate are produced in GBM cells, ROS begin to accumulate, resulting in mitophagy or autophagy (Yang et al. 2021). Furthermore, radiation has been shown to downregulate the system Xc‐antiporter in GBM cells, but this effect does not occur until about 48 h after treatment. At this point, the GBM cells become more vulnerable to excitotoxicity. (Yang et al. 2021).

### 3.3 mGlu3R Role in Glutamate Excitotoxicity

The mGlu3R receptor expressed in most GBM cell lines. Human glioblastoma cell line A172 has a limited effect on glutamate excitotoxicity as it possesses a negative feedback system through which it can inhibit adenyl cyclase activity and influence cellular proliferation. Activation of this receptor by glutamate starts a signaling cascade within the cell that leads to the inactivation of adenyl cyclase which decreases protein kinase B (Akt) activity. When inactivated this pathway allows adenyl cyclase to convert ATP to cAMP which activates the Akt pathway. This pathway produces brain‐derived neurotrophic factor (BDNF) that binds specifically to an NTRK2 receptor on Glioma Initiating Cells (GICs) which supports the growth of GICs. Therefore, antagonizing this mGlu3R with LY 341495 or LY 2389575 (two mGlu3R antagonists) would play a bigger role in GBM cell proliferation due to paracrine signaling. This would also help increase GBM cell sensitivity to TMZ (Ciceroni et al. 2013).

### 3.4 Modulation of GBM Tumor Invasiveness Through Glutamate Excitotoxicity

Invasive growth is a hallmark characteristic of GBM. Glutamate excitotoxicity is not only related to GBM cell growth and proliferation but also found to be linked to the invasive ability of GBM cells (So et al. 2021). In particular, the β1 integrin protein, which modulates the interaction between GBM cells, neuronal cells, and extracellular matrix, is significantly overexpressed in GBM cells. The activation of the β1 integrin protein leads to the formation of downstream focal adhesion complexes (FAK) which assists in the migration and invasion of GBM cells. Of note, β1 integrin protein is associated with the GluR1 subunit on AMPA receptors, which is activated by glutamate. It has previously been proposed that the AMPA receptor may play some role as a cytoskeleton anchor on GBM cells, assisting with further invasion (Corsi et al. 2019). Therefore, it is very likely that the microenvironment within GBM cells plays a large role in their invasive ability as an increased presence of glutamate may further drive the migration of GBM cells. (Corsi et al. 2019). This theory is further supported by a previous study which revealed that knockdown of GLuR1 in GBM cells inhibited cell invasion and proliferation (de Groot et al. 2008). Therefore, glutamate receptors, such as AMPA receptors, warrant further investigation as a potential target in GBM therapy.

Glutamate toxicity has also been implicated in the invasive ability of GBM cells through signaling by NMDA receptors, another glutamate‐dependent receptor that has emerged as a potential target to prevent GBM invasiveness. Activation of NMDA receptors promotes tumor growth, survival, and migration in GBM by enhancing matrix metalloproteinase‐2 (MMP‐2) activity that degrades the extracellular matrix components and facilitates glioma cell invasion (Ramaswamy et al. 2014). It has been studied that, extracellular matrix, Ca^2+^ signaling, and glutamate mediates invasion and migration in glioma cells (So et al. 2021). Glutamate released from glioma cells activate Ca²⁺‐ permeable AMPA and NMDA receptors, promoting cell migration and invasion. Prolonged Ca²⁺ influx triggers excitotoxic death of nearby neuronal and glial cells creating a microenvironment conducive to tumor invasion (So et al. 2021). Hence co‐targeting glutamate excitotoxicity and NMDA receptors can be considered as strategies for preventing GBM invasiveness.

According to this approach, it has been demonstrated that shRNA‐mediated targeted downregulation of system Xc (xCT or SLC7A11) reduces extracellular glutamate levels, leading to decreased invasion of GBM cells. The glutamate released from GBM cells, from excitotoxicity, acts as a signaling factor that promotes tumor invasiveness through metastatic abilities. This signaling activates protein kinases which phosphorylates AKT, stimulating cell proliferation. The glutamate also causes inflammation resulting from the activation of microglial cells surrounding the tumor (Sontheimer 2008). GLAST (Glutamate Aspartate transporter) also known as EAAT1 (Excitatory Amino Acid Transporter 1), is a membrane‐bound transporter primarily responsible for clearing extracellular glutamate. Interestingly, inhibiting GLAST expression has been found to limit the progression and invasion of GBM xenografts (Corbetta et al. 2019). This indicates that targeting GLAST may be a promising strategy to reduce tumor growth and invasion.

## 4 Biochemical Agents That Affect Glutamate Excitotoxicity in Glioblastoma

Many different biochemicals have been discovered to improve neuronal survival against high glutamate concentrations resulting from GBM. Tanshione is a Chinese herbal medicine commonly used to promote blood circulation. The sulfonic sodium derivative of this herb has been observed in SH‐SY5Y GBM cell lines to have an effect on glutamate excitotoxicity (Li et al. 2017). One of the mechanisms through which the herb accomplishes this is by reducing oxidative stress due to protein and lipid modification and the formation of ROS because of excessive glutamate accumulation within the cells. Tanshione also reduces glutamate‐induced neuronal apoptosis by regulating caspase proteases involved in apoptosis, specifically caspase‐3.

Another biochemical compound found to affect glutamate excitotoxicity are Sirtuin proteins (SIRT). These proteins are nicotinamide adenine dinucleotide (NAD)‐dependent histone deacetylases that help regulate metabolic functioning in every cell. There are seven different classes of SIRT, each carrying out various roles that help support cellular survival during stress. SERT4 in the A172 cell line was the only SIRT that directly affects glutamate excitotoxicity (Kunadis and Piperi 2022). It decreases the amount of glutamate released by GBM cells by inhibiting glutaminase, which converts glutamine to glutamate, and instead activates glutamate dehydrogenase, which redirects the intracellular glutamate towards the TCA cycle for utilization. This mechanism decreases calcium influx in proximal neurons because glutamate is required for calcium entry, thereby lowering the chance of hyperexcitability.

Furthermore, Astaxanthin (AST) is a natural antioxidant found within the body and can help modulate glutamate excitotoxicity by regulating proteins that favor hyperexcitability (Kandy et al. 2022). Although AST does not directly affect glutamate concentrations, it disrupts the signaling that occurs when glutamate binds to receptors. AST reduces intracellular calcium levels by downregulating the transcription of ionotropic kainate, AMPA, and NMDA receptors. This, in turn, decreases ROS production in neuronal cells exposed to glutamate (Kandy et al. 2022).

2,3,5,6‐tetramethylpyrazine (TMP), an alkylpyrazine compound, biosynthesized through acetoin amination is the primary component extracted from the Chinese herb Chuanxiong. Tetramethylpyrazine is used in clinical practice in combination with other drugs. It helps in regulation of blood glucose and lipids, protection of liver and in reducing inflammation and vascular endothelial injury (Guo et al. 2016). Tetramethylpyrazine (TMP), is linked to glutamate excitotoxicity as TMP mechanistically downregulates kainate, AMPA, and NMDA receptors, lowering intracellular calcium levels. Tetramethylpyrazine was tested for therapeutic potential against gliomas. In cultured glioma cells, 50 μM TMP was found to significantly reduce glutamate‐induced increase in intracellular calcium level, a key feature of excitotoxicity that contributes to tumor progression and neuronal damage (Fu et al. 2008). In rats with brain‐transplanted gliomas, tetramethylpyrazine was found to inhibit tumor growth and extend overall survival (Fu et al. 2008). TMP disrupts CXCR4/SDF‐1 signaling pathway, in glioma C6 cells inhibiting glioma cell migration and angiogenesis (Cai et al. 2014). These findings suggest TMP bears the ability to suppress glioma progression and but also protect neurons from glioma‐induced glutamate excitotoxicity, highlighting its potential for treating malignant gliomas.

Inhibition of glutaminase has been reported to preferentially reduce the growth of IDH1 mutant glioma cells (Seltzer et al. 2010). In addition, another biochemical permeable to the blood‐brain barrier which can be utilized to regulate glutamate excitotoxicity in GBMs is ebselsen, a glutathione peroxidase (GPx) imitator (Azad et al. 2014; Sies 1993). Ebselen works as a allosteric inhibitor of glutaminase (GLS), decreasing the production of glutamate in proximal neurons. (Thomas et al. 2013). Previous studies have shown that ebselen is quite a potent inhibitor as it has a two times greater activity in comparison to other GLS inhibitors (Thomas et al. 2013). Furthermore, ebselen not only modulates glutamate excitotoxicity but also sensitizes GBM cells for apoptosis. Furthermore, ebselen promotes GBM apoptosis through the Fas complex by inducing the formation of the Fas‐mediated DISC, the formation of the Fas‐associated death domain (FADD), as well as the activation of caspase 8 (Thomas et al. 2013). In some other studies, ebselen has also been shown to have some effects in decreasing the production of TNFalpha‐induced pro‐inflammatory factors, including IL‐6, IL‐8, monocyte chemoattractant protein‐1 (MCP‐1), and cyclooxygenase (COX2) which can help mediate inflammation in GBM, (Tewari et al. 2009). Moreover, ebselen has been shown to induce DNA damage repair signaling in GBM cells, as well as to decrease ROS production through the TNFalpha complex in GBM cells (Tewari et al. 2009). Given that Ebselen affects both glutamate metabolism and induces apoptosis in GBM cells, its use could be a powerful tool in treating GBM tumors.

### 4.1 TNF Alpha and Glutamate Excitotoxicity in GBM

Tumor necrosis factor (TNF) is an effective biochemical and inflammatory cytokine released by cells in response to structural stress. When excess TNF is produced it upregulates glutaminase, the enzyme that converts glutamine to glutamate and inhibits the reuptake of glutamate into GBM cells (Takeuchi et al. 2006). Therefore, glutamate and TNF levels exhibit a positive correlation that is toxic to neurons. Anti‐TNF agents could be a potential therapeutic option for reducing the amount of glutamate affecting neurons in GBM patients (Clark and Vissel 2016). However, since TNF and epidermal growth factor receptor (EGFR) are linked in GBM cells where both are expressed, blocking both would provide a better outcome. The combination therapy of Afantinid (a second‐generation EGFR inhibitor) and Pomalidomide (TNF inhibitor) has been‐shown to cause a decrease in GBM cell growth (Luo et al. 2020).

**Figure 2:**
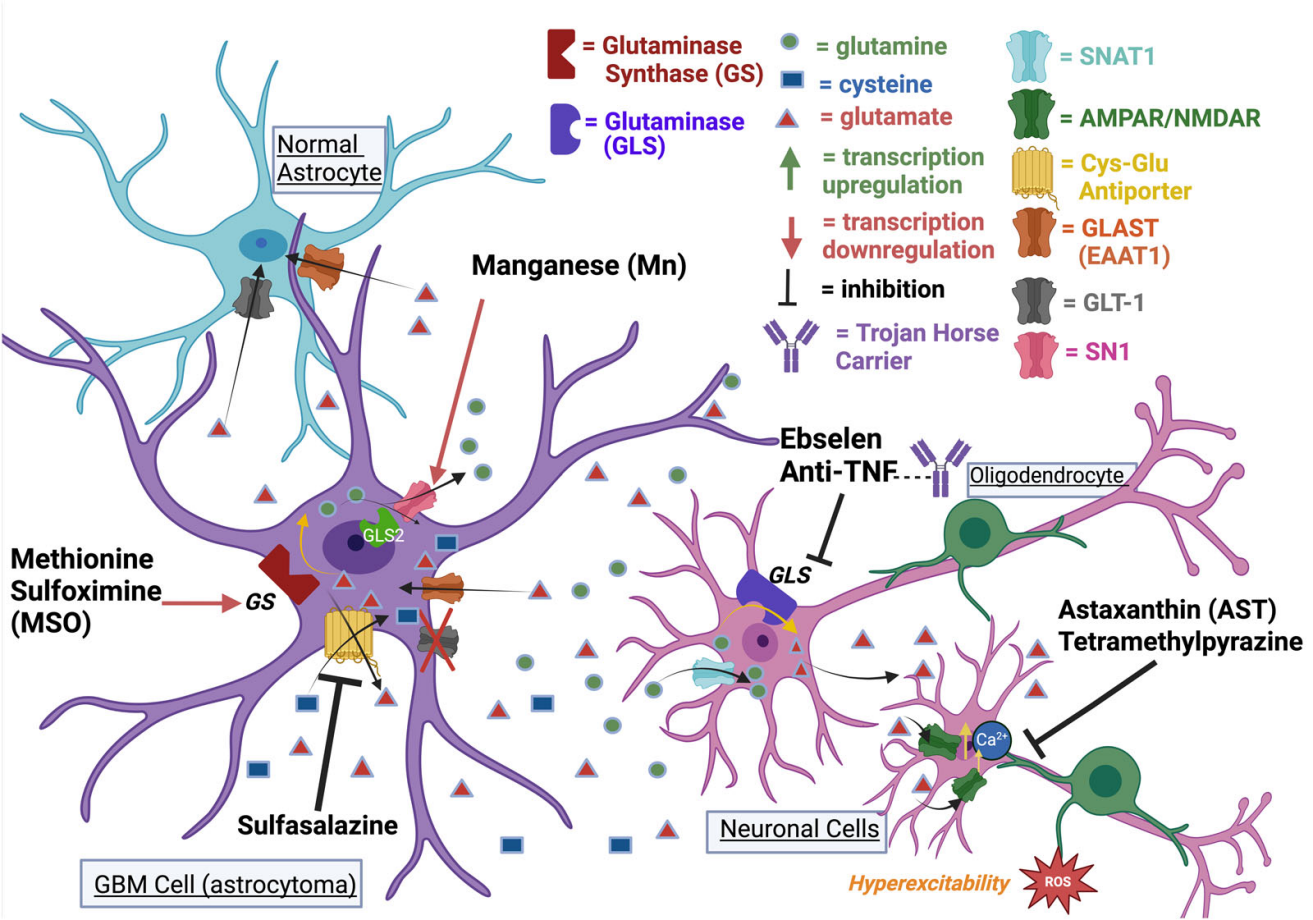
Biochemicals that can diminish the effect of glutamate excitoxicity in glioblastoma microenvironment by either targeting the glutamine‐glutamate cycle or the SXc antiporter. GLAST, glutamate‐aspartate transporter; GLT‐1, glutamate transporter 1; SN1, System N 1; SAT1, System A Transporter 1; AMPAR, α‐amino‐3‐hydroxy‐5‐methyl‐4‐isoxazolepropionic acid receptor; NMDAR, N‐methyl‐d‐aspartic acid or N‐methyl‐d‐aspartate receptor; Trojan Horse Carrier, IgG fusion protein that binds to anti‐TNF agents and a blood‐brain barrier receptor, allowing the agent to enter the brain. Created with BioRender.com

Although anti‐TNF would provide promising results, these inhibitors are impermeable to the blood‐brain barrier and would therefore need a carrier protein. A potential intervention to overcome this problem involves synthesizing these inhibitors as IgG antibodies attaching them to a molecule known as the ‘Trojan horse.’ This forms a complex that binds to a membrane receptor allowing anti‐TNF to cross the blood‐brain barrier. An example of a fusion protein utilized is Human Insulin Receptor Monoclonal Antibody (HIRMAb) (Pardridge 2010). Another therapeutic strategy could be delivering anti‐TNF agents using the Trojan horse molecules into the brain of IDH1 mutant GBM patients (Figure 2). Such therapeutic interventions may effectively reduce glutamate excitotoxicity by inhibiting the glutaminase enzyme. Lastly, methionine sulfoximine (MSO) is a biochemical that helps reduce glutamate excitotoxicity. It works by inhibiting glutamine synthetase (GS), downregulating the gene responsible for producing this enzyme (Martins et al. 2020). Although this would allow glutamate to accumulate within glioblastoma cells, it would reduce the amount of glutamine released, reducing glutamate excitotoxicity through the glutamine‐glutamate cycle. Additionally, the alkylating agent sulfasalazine inhibits the glutamate‐cysteine antiporter making it a potent sensitizer of GBM cells particularly when used in combination with methionine sulfoximine (Figure 2). This compound is more effective for glutamine‐independent GBM cell lines and demonstrates efficacy in low concentrations. However, it has been reported to cause neurotoxicity and should therefore be monitored closely if used in patients.

In summary, these biochemicals play a significant role in regulating glutamate excitotoxicity and could be combined as a potential adjuvant therapy for glioblastoma. Additionally, incorporating manganese, a naturally occurring element, with these biochemicals may further reduce glioblastoma cell survival. (Aschner et al. 2009). Manganese prevents glutamine deprivation in glioblastoma cells which prevents the upregulation of certain tumor suppressor genes such as p53, as previously described (Aschner et al. 2009). Figure 2 summarizes some alternative biochemicals and their mechanisms of action through which they can reduce glutamate excitotoxicity in glioblastoma microenvironment. A list of these biochemicals with their targets and their mechanisms of action in regulating glutamate excitotoxity are compiled in Table 2.

**Table 2:** Summary of biochemicals that can affect glutamate excitotoxity in glioblastoma and their associated mechanisms of action.

Biochemical	Target	Mechanism of Action
Tanshione	Reactive oxygen species (ROS) and neuronal apoptosis	Reduces oxidative stress and ROS production Reduces caspase protease induced neuronal apoptosis
Sirtuin proteins (SIRT)	Glutaminase	Inhibits glutaminase, reducing glutamate production Activates glutamate dehydrogenase, redirecting glutamate for utilization in TCA cycle
Astaxanthin (AST)	Lowers intracellular Ca^2+^ levels	Downregulates transcription of ionotropic kainate, AMPA, and NMDA receptors, reducing intracellular calcium levels and neuronal ROS production
Ebselen	Glutaminase	Inhibits glutaminase, reducing glutamate production Sensitizes GBM cells for apoptosis through the Fas complex
Anti‐TNF	Tumor necrosis factor (TNF)	By reducing TNF it inhibits glutaminase, reducing glutamate production
Methionine sulfoximine (MSO)	Glutamine synthetase	Inhibits glutamine synthase, reducing glutamine production and glutamate release through the glutamine‐glutamate cycle
Sulfasalazine	Cystine‐glutamate antiporter	Inhibits the cystine‐glutamate antiporter, reducing glutamate efflux
Tetramethylpyrazine	Lowers intracellular Ca^2+^ levels	Downregulates transcription of ionotropic kainate, AMPA, and NMDA receptors, lowering intracellular calcium levels and neuronal ROS production
Manganese	Activates SN1	Prevents glutamine deprivation in GBM cells

### 4.2 Role of IDH Wild‐Type and Mutant GBM in Glutamate Excitotoxicity

Isocitrate dehydrogenase (IDH) is a crucial enzyme involved in Kreb's Cycle which catalyzes the conversion of isocitrate to alpha‐ketoglutarate. However, a mutant type of IDH that is found in some GBM cell lines produces D‐2‐hydroxyglutarate (D‐2‐HG), which influences the production of glutamate as well as the expression of DNA repair enzymes genes in GBM cells through epigenetic mechanisms (Han et al. 2020). The IDH mutations in both mutant types (IDH1 and IDH2 mutants) that have been studied in current literature results in the substitution of the arginine (arginine 132 in IDH1 mutant and arginine 172 or 140 in IDH2 mutant) residue for another amino acid, typically a histidine residue. This results in the production of D‐2‐HG instead of α‐ketoglutarate. The accumulation of D‐2‐HG in this mutant type impacts glutamate excitotoxicity by inhibiting BCAAs transaminases (BCAAT) which is the enzyme that catalyzes the transformation of branched‐chain amino acids into glutamate (McBrayer et al. 2018). Therefore, patients with glioblastoma tumors containing mutant IDH are expected to have a longer survival compared to those with wild‐type IDH. This is because the mutant cells release lower concentrations of glutamate and have compromised repair enzymes. Moreover, glutamate depletion in GBM cells inhibits cell growth, as glutamate depletion results in the generation of reactive oxygen species (ROS) in GBM cells (Shi et al. 2014). Targeting the glutamine‐glutamate cycle, such as by inhibiting glutaminase, could prevent glutamate excitotoxicity and disrupt tumor growth by reducing glutathione production (Han et al. 2020).

## 5 Effect of Ketogenic Diet on Glutamate Excitotoxicity

The ketogenic diet (KD) provides little to no carbohydrate intake, focusing on fat and protein intake as the focus. Tumors often utilize excessive amounts of glucose and produce lactate even in the presence of oxygen, known as the Warburg effect. GBM cells have been reported to rely on this effect to maintain their energy stores, creating an acidic microenvironment (R. Zhang et al. 2023). When in the state of ketosis from the ketogenic diet, the liver produces 3‐hydroxybutryate and acetoacetate from fatty acids, also known as ketone bodies. When metabolized, ketone bodies are converted to acetyl‐CoA by citrate synthetase. This process reduces the amount of oxaloacetate available, and this blocks the conversion of glutamate to aspartate. As a result, glutamate is instead converted into GABA, an inhibitory neurotransmitter, by the enzyme glutamate decarboxylase (Yudkoff et al. 2007). Therefore, this diet‐induced reduction of glutamate has potential in reducing the adverse effects of GBM‐induced glutamate excitotoxicity.

Additionally, a key point is that a ketogenic diet can decrease extracellular glutamine levels by increasing leucine import through the blood‐brain barrier, thereby reducing glutamate production via the glutamine‐glutamate cycle. (Yudkoff et al. 2007). The potential to reduce glutamate excitotoxicity may be an underlying metabolic mechanism that makes the ketogenic diet a promising inclusion in the therapeutic approach for GBM.

A ketogenic diet has also been shown to lower levels of tumor necrosis factor‐alpha (TNF‐α) in mice (Dal Bello et al. 2022). This reduction in tumor necrosis factor alpha (TNF‐α), a major regulator of inflammatory responses, may benefit glioblastoma patients by decreasing glutamate release from GBM cells, given the positive correlation between glutamate and TNF‐α (Clark and Vissel 2016). Furthermore, utilizing a ketogenic diet as a way of reducing glioblastoma inflammation and growth might serve as a more affordable intervention to slow the tumor growth which might enhance the effectiveness of conventional treatments like radiation and chemotherapy.

## 6 Conclusion

Glutamate excitotoxicity is the primary mechanism by which GBM cells induce neuronal death, creating more space for tumor expansion in the brain. Our literature review emphasizes that this process is essential for the growth of GBM tumors, as it provides glioblastoma stem cells with the necessary metabolic fuel for continued proliferation. Glutamate excitotoxicity occurs mainly through the SXc antiporter system but can also result from the glutamine‐glutamate cycle. Targeting both the antiporter system and the cycle may reduce glutamate exposure to neurons, providing a therapeutic benefit and potentially improving glioblastoma patient survival.

This review highlights the key sources of glutamate excitotoxicity driven by GBM cells and identifies signaling pathways that may serve as therapeutic targets to control glioblastoma proliferation, growth, and prognosis. Future research should focus on developing targeted and pharmacological interventions to regulate glutamate production and inhibiting glutamate‐generating pathways within glioblastoma tumors to improve patient outcomes.

## Author Contributions

**Colin Moriarty and Debanjan Bhattacharya:** Involved in writing and reviewing the draft, preparing figures and revisions. **Natasha Gupta:** assisted in writing and reviewing draft and preparing revisions. **Debanjan Bhattacharya:** conceptualization, overall supervision, writing and reviewing draft and preparation of figures and revised versions of the manuscript. All authors have read and agreed to the published version of the article.

## Ethics Statement

The authors have nothing to report.

## Conflicts of Interest

The authors declare no conflicts of interest.

## Data Availability

The authors have nothing to report.
